# Genomes and phenomes of a population of outbred rats and its progenitors

**DOI:** 10.1038/sdata.2014.11

**Published:** 2014-06-10

**Authors:** Amelie Baud, Victor Guryev, Oliver Hummel, Martina Johannesson, The Rat Genome Sequencing and Mapping Consortium, Amelie Baud, Victor Guryev, Oliver Hummel, Martina Johannesson, Roel Hermsen, Pernilla Stridh, Delyth Graham, Martin W McBride, Tatiana Foroud, Sophie Calderari, Margarita Diez, Johan Ockinger, Amennai D Beyeen, Alan Gillett, Nada Abdelmagid, Andre Ortlieb Guerreiro-Cacais, Maja Jagodic, Jonatan Tuncel, Ulrika Norin, Elisabeth Beattie, Ngan Huynh, William H Miller, Daniel L Koller, Imranul Alam, Samreen Falak, Mary Osborne-Pellegrin, Esther Martinez-Membrives, Toni Canete, Gloria Blazquez, Elia Vicens-Costa, Carme Mont-Cardona, Sira Diaz-Moran, Adolf Tobena, Diana Zelenika, Kathrin Saar, Giannino Patone, Anja Bauerfeind, Marie-Therese Bihoreau, Matthias Heinig, Young-Ae Lee, Carola Rintisch, Herbert Schulz, David A Wheeler, Kim C Worley, Donna M Muzny, Richard A Gibbs, Mark Lathrop, Nico Lansu, Pim Toonen, Frans Paul Ruzius, Ewart de Bruijn, Heidi Hauser, David J Adams, Thomas Keane, Santosh S Atanur, Tim J Aitman, Paul Flicek, Tomas Malinauskas, E Yvonne Jones, Diana Ekman, Regina Lopez-Aumatell, Anna F Dominiczak, Rikard Holmdahl, Tomas Olsson, Dominique Gauguier, Norbert Hubner, Alberto Fernandez-Teruel, Edwin Cuppen, Richard Mott, Jonathan Flint, Jonathan Flint

**Affiliations:** 1 EMBL-EBI, Wellcome Trust Genome Campus, Hinxton, Cambridgeshire, CB10 1SD, UK; 2 European Research Institute for the Biology of Ageing, Rijksuniversiteit Groningen, Universitair Medisch Centrum Groningen, Groningen, 9700 AD, The Netherlands; 3 Max-Delbruck Center for Molecular Medicine, Berlin, 13125, Germany; 4 Department of Medical Biochemistry and Biophysics, Division of Medical Inflammation Research, Karolinska Institutet, Stockholm, SSE-17177 Sweden; 5 Wellcome Trust Centre for Human Genetics, Roosevelt Drive, Oxford OX3 7BN, UK; 6 Hubrecht Institute, Koninklijke Nederlandse Akademie van Wetenschappen and University Medical Center Utrecht, Utrecht, The Netherlands; 7 Neuroimmunology Unit, Department of Clinical Neuroscience, Centre for Molecular Medicine, Karolinska Institutet, Stockholm, Sweden; 8 British Heart Foundation (BHF) Glasgow Cardiovascular Research Centre, Institute of Cardiovascular & Medical Sciences, Glasgow University, Glasgow, UK; 9 Department of Medical and Molecular Genetics, Indiana University School of Medicine, Indianapolis, Indiana, USA; 10 Institut National de la Santé et de la Recherche Médicale (INSERM) Unité Mixte de Recherche Scientifique (UMRS) 872, Cordeliers Research Centre, Paris, France; 11 Department of Orthopedic Surgery, Indiana University School of Medicine, Indianapolis, Indiana, USA; 12 INSERM U698, Hôpital Bichat, Paris, France; 13 Medical Psychology Unit, Department of Psychiatry & Forensic Medicine, Institute of Neurosciences, Universitat Autònoma de Barcelona, Bellaterra, Barcelona, Spain; 14 Commissariat à l’Energie Atomique, Institut de Génomique, Centre National de Génotypage, Evry, France; 15 Department of Computational Biology, Max Planck Institute for Molecular Genetics, Berlin, Germany; 16 Pediatric Allergology, Experimental and Clinical Research Center, Charité Universitätsmedizin Berlin, Berlin, Germany; 17 Human Genome Sequencing Center, Baylor College of Medicine, Houston, Texas, USA; 18 The Wellcome Trust Sanger Institute, Hinxton, Cambridge, UK; 19 Physiological Genomics and Medicine Group, Medical Research Council Clinical Sciences Centre, Faculty of Medicine, Imperial College London, Hammersmith Hospital, London, UK; 20 Division of Structural Biology, Wellcome Trust Centre for Human Genetics, University of Oxford, Oxford, UK; 21 DZHK (German Centre for Cardiovascular Research), Partner site Berlin, Berlin, Germany

## Abstract

Finding genetic variants that contribute to phenotypic variation is one of the main challenges of modern genetics. We used an outbred population of rats (Heterogeneous Stock, HS) in a combined sequence-based and genetic mapping analysis to identify sequence variants and genes contributing to complex traits of biomedical relevance. Here we describe the sequences of the eight inbred progenitors of the HS and the variants that segregate between them. We report the genotyping of 1,407 HS rats, and the collection from 2,006 rats of 195 phenotypic measures that are relevant to models of anxiety, type 2 diabetes, hypertension and osteoporosis. We make available haplotype dosages for the 1,407 genotyped rats, since genetic mapping in the HS is best carried out by reconstructing each HS chromosome as a mosaic of the progenitor genomes. Finally, we have deposited an R object that makes it easy to incorporate our sequence data into any genetic study of HS rats. Our genetic data are available for both Rnor3.4 and Rnor5.0 rat assemblies.

## Background & Summary

Uncovering genetic variants that contribute to variation in complex traits is expected to provide insights into the biology of these traits. Genetic mapping in humans and animal models has identified many regions of the genome that contribute to variation in quantitative traits (Quantitative Trait Loci, QTL), but has been less successful at revealing causal variants^1–3^. Finding causal variants would allow a mechanistic understanding of how phenotypic variation arises, and aid with the identification of relevant genes.

**Figure 1:**
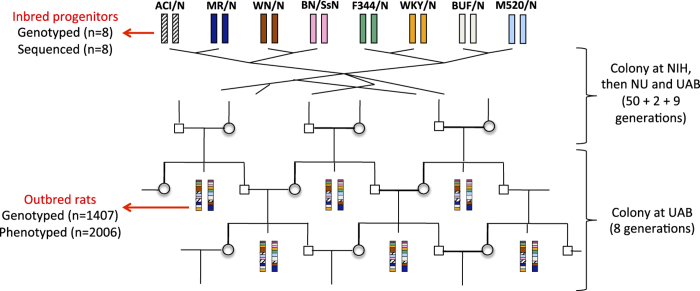
Experimental design and data collected. The Heterogeneous Stock is descended from eight inbred progenitors through more than 60 generations of outbreeding. As a result, each HS rat chromosome is a fine-grained mosaic of the founder genomes. The outbred rats included in this study were from generations 62 to 70 and were bred at the University of Barcelona (UAB). NIH: National Institute of Health; NU: Northwestern University (see methods). The data collected are shown on the left hand side of the figure, with the number of observation for each indicated in brackets.

In a study published in Nature Genetics^4^, we investigated the use of sequence information to find the sequence variants and genes responsible for phenotypic variation. We used an outbred population of rats descended from eight inbred progenitors (ACI/N, BN/SsN - a sub-strain of the reference strain BN, BUF/N, F344/N, M520/N, MR/N, WKY/N and WN/N) through more than 60 generations of outbreeding^5,6^ (Figure 1). The Heterogeneous Stock (HS) was chosen for its potential for high-resolution mapping. Because a large number of recombination events have accumulated over the generations, each HS rat is a fine-grained mosaic of the founder genomes.

The known ancestry of the HS offers an additional advantage: by sequencing the eight progenitors only, it is possible to evaluate whether one or more causal variant(s) segregate(s) at each QTL mapped in the outbred rats, and when a single variant was likely to account for the QTL, sequence information allowed identifying the causal variant and/or gene at about 10% of the QTLs. Our results provided insights on models of anxiety, type 2 diabetes, osteoporosis and the cardiovascular function (Table 1).

**Figure 2:**
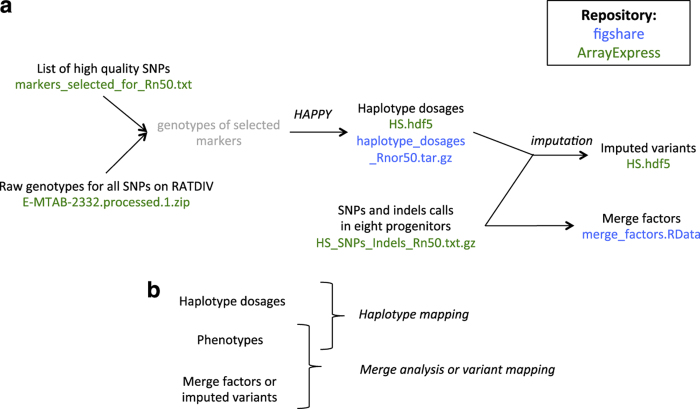
Generation and use of derived genetic data. (**a**) This flow chart shows how the derived genetic data (haplotypes dosages, merge factors, imputed variants) were obtained from the raw data. The name of the file corresponding to each data item is shown, and coloured in green when it was submitted to ArrayExpress (Data Citation 1), and blue when it was submitted to figshare (Data Citation 2). (**b**) Data to combine for genetic mapping and merge analysis.

We collected 195 phenotypes of biomedical relevance (Supplementary Table 1) on 2,006 outbred rats, and genotyped both 1,407 of the outbred rats and the eight progenitors (Figure 1) using a custom Affymetrix array (see supplementary note in ref. 4 for more information on the array). Because the outbred rats are descended from more than two progenitors, genetic mapping in the HS is best carried out by testing for association between the phenotype and the progenitor haplotypes^4,7^ rather than the genotypes. Therefore, we reconstructed each HS rat chromosome as a mosaic of the founder haplotypes using the HAPPY software^7^. We also sequenced the eight progenitors of the population (Figure 1) with SOLiD technology in order to investigate causal variants using a statistical method called merge analysis^8^. Figure 2 shows how the HS genotypes and progenitor sequences can be combined for different analyses. We submitted both raw data (phenotypes and genotypes of the outbred rats, sequences of the progenitors) as well as derived data (haplotype dosages for the outbred rats, sequence variants calls formatted for merge analysis) to ArrayExpress (Data Citation 1) and figshare (Data Citation 2). The raw data are available for both the previous Rnor3.4 and current Rnor5.0 rat assemblies while the derived data are available for the current assembly only.

The data collected on the outbred rats are specific to the animals used in this study, but they may be used for meta-analysis with data collected on other HS rats. The sequences of the progenitors and the resulting variant calls will be invaluable for those investigators that have phenotyped and genotyped other HS rats (or plan to do so)^9–11^, and in fact for any genetic study of a cross descended from some of the HS progenitors. Finally, combined with other rat sequences^12^, our data may also support population genetics studies.

## Methods

### Animals

The rat Heterogeneous Stock was established in the 1980s at NIH (Dr Carl Hansen) and went through 50 generations of rotational breeding there. A colony was established at Northwestern University, Chicago, IL (Dr Eva Redei) where the Stock went through another 2 generations before 40 breeding pairs were sent to the University Autonomous of Barcelona (UAB, Dr Alberto Fernandez-Teruel) where the rats used for this study were bred using a rotational breeding scheme (further details are available in ref. 6). The Stock went through another 9 generations at UAB before the first rats were genotyped and phenotyped. The scheme used to produce HS rats for phenotyping while maintain the colony is illustrated in Figure 1. At each generation, one male and one female from each sibship were kept to breed the next generation while a maximum of 8 of the remaining siblings were included in the study, keeping half of each sex whenever possible. Animals were housed in pairs (males) or groups of three (females), in macrolon cages (50×25×14 cm), and maintained with food (standard diet A04 by Panlab L.S Barcelona, Spain and tap water available ad lib, under conditions of controlled temperature (22±2 °C; relative humidity 50–70%) and a 12-h light-dark cycle (lights on at 08:00 h). Rats from eight generations are represented in the dataset, phenotyped over a period of three years.

All procedures were carried out in accordance with Spanish legislation on the Protection of Animals Used for Experimental and Other Scientific Purposes and the European Community's Council Directive (86/609/EEC) on this subject. The experimental protocol was approved by the Autonomous University of Barcelona ethics committee (permit CEEAH 697).

### Phenotyping

The phenotyping pipeline is presented in Table 1. The phenotypes were uploaded to a database (Integrated Genotyping System^13^) as they were collected. A description of all the measures (phenotypes and covariates) collected is given in Supplementary Table 1. All the below phenotyping protocols are a more detailed description of the methods published in the Nature Genetics paper^4^.

### Coat colour

The following code is used to describe coat colour: DB (dark brown), DBsp (dark brown, spotted), LB (light brown), LBsp (light brown, spotted), W (white).

### Fear related behaviours

All testing was performed between 9 h and 19 h. For each behavioural test males and females, as well as families, were tested in a counterbalanced manner across the time of the day and across days. Behavioural testing is included in *Whole organism phenotyping* in Supplementary Table 2.

#### Elevated zero maze:

The first behavioural test was the elevated zero maze, which is used to evaluate unlearned anxiety-like behaviour in rodents^14–17^. The maze, similar to that described by Shepherd *et al.*, comprised an annular platform (105 cm diameter; 10 cm width) made of black plywood and elevated to 65 cm above the ground level. It had two open sections (quadrants) and two enclosed ones (with walls 40 cm height). The apparatus was situated in a black testing room, dimly illuminated with red fluorescent light (approximately 50 lux at the level of the apparatus). The rats were individually placed in an enclosed section facing the wall and the behaviour was videotaped and measured outside the testing room^15^. The apparatus was cleaned with 20% ethanol and dried with paper towel between consecutive rats. The measures collected in the zero maze are: latency to enter an open section, time spent in the open sections, number of entries in the open sections, number of times the rat went from an open to a closed section or *vice versa*, number of head dips, and number of stretched attend postures.

#### Automated novel-cage ‘open field-like’ activity:

Exploration of a novel, open field-like environment (i.e., the ‘novel-cage’ activity test), has been traditionally considered as related to fearfulness (i.e., the lower exploration, the higher the level of fearfulness), a contention which is also supported from previous work showing associations between activity during 5 minutes in the novel cage and typical anxiety responses in the light-dark test and the elevated zero-maze test^15–17^. If longer testing intervals are used, the test reflects general activity (i.e., activity with lesser influence from novelty) in both mice and rats. The apparatus (Panlab, Barcelona, Spain) consisted of a horizontal surface (50×50 cm) provided with photo beams that detect movement and measure it automatically, loading the data in a computer. The rats were placed in transparent Plexiglas cages (40×40×40 cm) facing the wall, in a white fluorescent (60 w) illuminated chamber^15^, and their movements recorded for 30 min. There were 4 identical activity cages separated by an opaque plate (to avoid vision among rats from different cages), thus 4 rats were tested simultaneously and individually. Each activity cage was cleaned with 20% ethanol and dried with paper towel between consecutive rats. Activity level during the first 5 min indicates (open field-like) response to novelty while activity in the last 5-min intervals is indicative of habituated exploration^15,16^. The measures collected in the novel cage are: number of boli, distance travelled and number of rearings (upper beam breaks) during different intervals.

#### Two-way active avoidance acquisition and context-conditioned fear in the shuttlebox:

The acquisition of two-way active avoidance in a shuttlebox is an aversive learning task and a well-validated measure of conditioned anxiety, which is known to be mediated by a ‘passive avoidance/active avoidance’ conflict^18^. Context-conditioned freezing, indicating conditioned fear, can be measured during the initial inter-trial intervals of the first few trials of the shuttlebox avoidance task (i.e., before the first avoidance response^17^). Two-way active avoidance sessions were performed in three identical shuttleboxes (Letica Instr., Panlab, Barcelona), each one placed in independent sound-attenuating boxes constructed of plywood. A dim and diffuse illumination was provided by a fluorescent bulb placed behind the opaque wall of the shuttle boxes, which gave approximately 50-lux intensity inside each of the two compartments of the shuttle boxes. The experimental room was kept dark. The shuttleboxes consisted of two equally sized compartments (25×25 cm, 28 cm) connected by an opening (8×10 cm). Rats were allowed a 4-min period of familiarization to the box. Immediately after that period, a 40-trial session/rat was administered, each trial consisting of a 10-s CS (conditioned stimulus; 2400 Hz, 63-dB tone plus a 7-W small light) followed after termination by a 20-s US (unconditioned stimulus; scrambled 0.7 mA foot shock) delivered through the grid floor. Each shuttlebox was carefully cleaned with 96% ethanol and dried with paper towel between consecutive rats. Crossings to the other compartment during the CS (avoidances) or US (escapes) switched off the stimuli and were followed by a 60-s inter-trial interval. Context-conditioned freezing (i.e., classically-conditioned fear) was measured by two trained observers as the time a rat spent completely motionless except for breathing movements during the 60-min inter-trial intervals of the first 5 training trials ^6,15–17^. The measures collected in the shuttlebox are: number of avoidances, latency to avoid, number of crossings from one compartment to the other during the habituation period, number of crossings between the trials, number of freezings and time spent freezing.

### Intraperitoneal Glucose Tolerance Test (IPGTT)

Food was removed in the morning and tests were carried out between 11am and 12 noon. Conscious rats in the post absorptive state were injected intraperitoneally with a solution of glucose (Sigma D9559, 2 g/kg body weight). Blood samples were collected by tail tipping (<1 mm of the end of the tail) in unrestrained rats. Blood glucose was read before glucose injection and 30, 60 and 120 min afterwards. Blood glucose concentration was determined using a glucose meter (Accucheck, Roche Diagnostics, Welwyn Garden City, UK). Cumulative glycemia (AUC_G) was calculated as the increment of the values of plasma glucose during the IPGTT. Incremental plasma glucose values above baseline integrated over 120 min, after an injection of glucose, were used to calculate the index of glucose tolerance (DeltaG). IPGTT is included in *Whole organism phenotyping* in Supplementary Table 2.

### Blood pressure

Systolic blood pressure was measured by tail plethysmography in conscious, restrained animals as previously described^19,20^. In short, the rats were pre-warmed for 15 min at 30 °C for tail artery vasodilation. Warming was carried out using a ventilated polystyrene box and an overhead light source. Rats were then wrapped in a cloth for restraint, with no prior training, and an inflatable cuff placed on their tail along with a piezoceramic transducer (Hartmann & Braun type 2). Pulse detection was visualised as a function of pressure and displayed using Microsoft Windows compatible software. 6–8 pressure readings were taken for each rat on a single day, and averaged. Blood pressure measurement is included in *Whole organism phenotypin*g in Supplementary Table 2.

### Basal hematology

A full blood count was used to characterise haematological parameters. Rats were anaesthetized with isofluorane (O_2_ flow of 2 L/min with 3–5% isofluorane) and placed in ventral position. While holding tail at 135° angle, a cut, 3–5 cm from the tip of the tail, was made across the ventral side with a scalpel. Blood (300 μl/sample) was collected from the lateral tail vein of anesthetized rats into a tube pre-treated with the anticoagulant EDTA-2K (Sangüesa) and inverted 10 times. Blood was collected immediately after the rats were injected with emulsified myelin oligodendrocyte glycoprotein (MOG, used to induce experimental autoimmune encephalomyelitis—see below), which therefore will not have affected basal hematology. The blood was stored at 4 °C for up to 12 h until analysed. The samples are identified as *blood_basal_hematology* in Supplementary Table 3.

Full blood count measures were acquired by an automatic hemocytometer (ADVIA 120 Hematology analyzer from Bayer, Siemens Diagnostics). This assay is identified by the method *Blood count* in Supplementary Table 2.

### Basal immunology

Fluorescence-activated cell sorting (FACS) was used to get the basal immunology profile of the rats. Blood was collected at the same time as the haematology samples above into a 1.5 ml polypropylene tube (Eppendorf Safe-Lock) pre-filled with 4 μl (20 units) heparin (Leo Pharma) and inverted immediately at least 10 times and stored at 4 °C until used (1–10 h). The samples are identified as *blood_basal_immunology* in Supplementary Table 3.

Twenty microliter of blood was added in duplicate to 96 well v-bottom polypropylene plates (BD Falcon), taking care to not include any coagulated samples. Up to five different fluorescently labelled or biotin-conjugated monoclonal antibodies (MAbs; see below) were diluted in FACS-buffer (Mg^2+^ and Ca^2+^ free PBS-D [Life Technologies, Carlsbad, CA, USA] containing 1% bovine serum albumin [Sigma-Aldrich], 2 mM Na_2_EDTA [Merck, Darmstadt, Germany] and 0,02% NaN_3_) to pre-determined optimal concentrations and combined into a single tube. Antibody concentrations were considered optimal if negative and positive populations were sufficiently separated or, for the measurement of mean fluorescence intensity, when the antibody had reached a saturating level. A corresponding volume of 2 μl per antibody was added per well containing non-lysed whole blood. Aliquots from several samples were pooled and used for compensation set-up and fluorescence minus one (FMO) controls. These controls were added on a separate plate. Samples were mixed for 5 s by gentle vortexing, placed on ice in a Styrofoam box, covered with aluminium foil and allowed to incubate for 20 min. After incubation, a total volume of 300 μl FACS-buffer was added per well followed by centrifugation at 485×*g* for 3 min (4 °C). The supernatant was discarded and fluorochrome-conjugated streptavidin was added at 2 μl per well, followed by incubation for 15 min on ice. Without further washing, 220 μl of a hypertonic buffer containing 8.29 *g* NH4Cl (Sigma-Aldrich), 1.0 g KHCO_3_ (Sigma-Aldrich) and 37.2 mg Na_2_EDTA (Merck), pH 7.2 per dm^3^ was added per well. Samples were allowed to incubate at room temperature for 10 min or until lysis was complete in all wells. After centrifugation, samples were washed once in hypertonic buffer and thereafter incubated for 15 min in 220 μl of 0.5% (w/v) phosphate-buffered formaldehyde solution (Sigma-Aldrich) at room temperature, taking care to mix the cells thoroughly before the incubation. Remaining fixative was removed by washing the cells twice in FACS-buffer. Stained cells were kept for up to six days in FACS-buffer in the dark until analysed. Duplicate samples were pooled before acquisition, which was performed on a four-colour BD FACS Calibur for the first four batches and thereafter on a BD SORP LSR-II Analytic Flow Cytometer. The data was analysed with FlowJo (Tree Star Inc., Ashland, OR).

Antibodies used for flow cytometry: The following MAbs were purchased from BD Pharmingen (San Diego, CA): CD45 (OX-1 FITC), RT1-B (OX-6 PE), CD45RA (OX-33 Pe-Cy5), CD45RC (OX22-PE), αβTCR (R73 biotin and PerCP/APC), CD8a (OX-8 FITC), CD25 (OX-39 PE/APC), CD4 (OX-35 APC/Pe-Cy5), CD28 (JJ319 FITC), pan-MHC class I (OX-18 PE), pan-granulocyte (HIS-48 biotin/Fitc). Streptavidine (SA)-APC was used as a secondary reagent. Cell surface expression of a molecule (e.g., CD25) was measured as the mean fluorescence intensity (MFI) of the corresponding staining. The subset of cells with highest expression was identified as ‘high’ (e.g., CD25highCD4) and their frequency in a particular population of cells was calculated as percent of the parent population (e.g., CD4 T cells). Subsets of white blood cells (WBCs) were determined by multiplying the proportion of that particular subset (e.g., B cells) with the total CD45+ population (CD45 is ubiquitously expressed on all WBCs). The concentration of WBCs in the blood was then determined using a hemocytometer (basal hematology measure). This assay is identified by the method *FACS* in Supplementary Table 2.

### Experimental Autoimmune Encephalomyelitis (EAE)

EAE was induced by injecting myelin oligodendrocyte protein emulsified with Freund’s adjuvant subcutaneously at the base of the tail. This injection was performed immediately before tipping the tail of the rats to collect blood for basal immunology and basal hematology. Therefore, those measures should not be affected by the injection. The rats were then scored daily as follows: 0=healthy; 1=tail weakness or tail paralysis; 2=hind leg paresis or hemiparesis; 3=hind leg paralysis or hemiparalysis; 4=tetraplegy, urinary, and/or fecal incontinence; and 5=death. If severe disease (score 4) was observed for two consecutive days, the rats were sacrificed for ethical reasons. The daily scores are not provided as part of this Data Descriptor, but measures capturing the severity of EAE are provided so that an effect of the disease on the other phenotypes can be tested. These measures are: whether the rat died of EAE (possibly sacrificed for ethical reasons), maximum score between injection and sacrifice, and score at sacrifice.

### Euthanasia and tissue dissection

Unfasted rats were euthanized between 10am and 4pm by exsanguination under isofluorane anesthesia (O2 flow (2 L/min) with 3–5% isofluorane). Blood was obtained by cardiac puncture, then the heart was dissected out, weighed and snap-frozen. Thereafter the punched ear, abdominal aorta, liver and bones were dissected in parallel whenever possible. Treatment of the ear, aorta and bones are detailed in the following subsections. Liver samples were snap-frozen. Other tissues (thymus, brain, pituitary, spinal cord, spleen, kidneys, adrenal glands, tail) were collected and snap-frozen for future studies, but no data are submitted as part of this Data Descriptor from these samples.

### Serum biochemistry

Blood was collected by a heart puncture in the left ventricle with a 23-gauge needle. Blood was kept at room temperature for 4 h, then at 4 °C for 4–12 h until it was centrifuged at 2000 rpm for 20 min. The serum was separated, aliquoted and stored at −80 °C. The samples are identified as *serum* in Supplementary Table 3.

Serum was analysed on a fully automated Olympus AU400 chemistry Immuno Analyser at the MRC Harwell laboratory. This assay is identified by the method *Biochemical analysis* in Supplementary Table 2.

### Arterial elastic lamina ruptures

The spontaneous rupture of the internal elastic lamina (IEL) in various arteries occurs to different extents in different rat strains. In the Brown Norway rat, large numbers occur in the abdominal aorta (AA) and common iliac arteries (IA) and so this rat strain is a model of large artery elastic lamellar defects, and thus indirectly, of interest for studying factors affecting aneurysm formation. We have quantified this phenomenon in the AA and in the left IA (LIA) in 1002 of the HS rats, from generation 5 to generation 8. To do this, the AA and the proximal 1 cm of the LIA were dissected out, rapidly rinsed in saline and fixed by immersion in buffered formalin. The samples are identified as *aorta* in Supplementary Table 3.

‘En face’ preparations of the unperfused AA and the attached LIA were then made. Under a dissecting microscope, arteries were cleaned, opened longitudinally and pinned out, luminal surface uppermost. The luminal surface was stained first with acid orcein (1% orcein in 100 mls of 70% ethanol,+0.6 mls of fuming HCl. Staining time 15 min.) and after washing, with Groat’s hematoxylin (1 g ferric ammonium sulfate dissolved in 50 ml distilled water+0.8 ml fuming H2SO4 mixed with 50 ml of 1% solution of hematoxylin in 95% ethanol. Staining time around 5 min.) to show the internal elastic lamina (IEL) and the nuclei respectively. Stains were applied drop by drop to the surface of the preparation so as not to stain the underside. After staining, arteries were dehydrated, unpinned, cleared and mounted on slides for microscopic observation. With this technique, as previously described^21^, ruptures appear as dark grey transverse bands due to absence of the internal elastic lamina, which stains pink, and the intense staining of underlying smooth muscle cell nuclei, which are not stained in areas where the IEL is present. Ruptures were then quantified at a final magnification of x 40. For each individual, the total number of IEL ruptures in the AA and LIA were recorded and each rupture was graded on a semi-quantitative scale according to its size in the circumferential direction, using a grid in the eyepiece. A final score was calculated taking into account the size of the ruptures. Thus the degree of IEL rupture in each AA and LIA was expressed as a number of ruptures per artery, or as a score indicating the severity of the phenomenon. For each rat, global values were also obtained by adding those for AA and LIA. This assay is identified by the method *Staining and observation under the microscope* in Supplementary Table 2.

### Bone mass and strength

At dissection, the bones were kept on ice until stored at −20 °C. The samples are identified as *bones* in Supplementary Table 3.

For bone density and structure, femurs were placed in plastic tubes filled with 70% ethyl alcohol and centered in the gantry of a Norland Stratec XCT Research SA+pQCT (Stratec Electronics, Pforzheim, Germany). Slice measurements of 0.26 mm thickness and a voxel size of 0.07 mm were taken at the midshaft, distal femur and perpendicularly through the femoral neck. For each slice, the X-ray source was rotated through 180° of projection. Volumetric BMD (vBMD; mg/cm3), cross sectional area (CSA; mm2) and polar moment of inertia (Ip; mm4) were measured from the pQCT images. Density thresholds of 500 and 900 were used to identify mineralized bone. The femur BMC (g) was measured using DXA (PIXImus mouse densitometer, Lunar Corp., WI, USA). The femoral neck width (NW; mm) was measured in the anterior-posterior direction using digital calipers. For bone biomechanics, femurs were tested in three-point bending by positioning them on the lower supports of a three-point bending fixture and applying load at the midpoint using a material testing machine (Alliance RT/5, MTS Systems Corp., Eden Prairie, USA). Force and displacement measurements were collected every 0.05 s. From the force versus displacement curves, work to failure (W; in mJ) was calculated in TestWorks software. This assay is identified by the method *Bone analysis* in Supplementary Table 2.

### Wound healing

The ear punch model was used to measure wound healing^22^. At 7 weeks of age a 2-mm hole was made in the centre of the cartilaginous part of one ear using a metal ear punch (Fine science tools, model: 24210/02). At the end of the experiment, the whole ear was cut out with scissor and placed in 1.5 ml 10% buffered formalin. The samples are identified as *ear* in Supplementary Table 3.

To calculate the area of the hole, the ear was first trimmed and blotted dry with some tissue so that it could be placed flat between two slides (we used VWR 72 Polysine or Superfrost with dimensions 25×75×1 mm), and scanned (48-bit colour, 4800 dpi). The hole was delimited and the area calculated using the software ImageJ. The scale was set by using the straight line tool across the side of the slide (25 mm) and ticking the ‘Global’ box, then the hole area was measured by using the free line drawing tool around the hole in the ear. This assay is identified by the method *Image analysis* in Supplementary Table 2.

### DNA extraction and genotyping

Approximately 50 mg of liver tissue (dissected from the outbred rats and kindly provided by Dr Myrna Mandel, NIH, for the eight progenitors) was subjected to DNA extraction using a classical Phenol/chlorofrom method (Sambrook, Fritsch, Maniatis) with a subsequent RNAse A digest. DNA was resolved in low EDTA buffer (10 mM Tris 0,1 mM EDTA pH 8.0) to a final concentration of 50 ng/μl. DNA extraction for all samples was performed at the Max-Delbrück Center for Molecular Medicine, MDC, Berlin, Germany.

Genotyping was carried out using a custom Affymetrix array (RATDIV array; see supplementary note in ref. 4 for more information on the array). Briefly, the array interrogates 805,399 SNPs chosen based on partial sequence data available for 14 rat strains, three of which are amongst the HS founder strains (Brown Norway BN, Fischer F344 and Wistar Kyoto WKY). Work by the STAR consortium had shown that variation segregating between these 14 strains is representative of that in most other laboratory strains^23^, so it was expected that the RATDIV array would be appropriate for genotyping the Heterogeneous Stock.

Genotyping was performed according to the Affymetrix SNP chip 6.0 protocol using 250 ng genomic DNA for StyI and NspI, respectively. Genotyping was performed either at the MDC or at the Centre National de Genotypage (CNG), Evry, France. This information is included in the metadata submitted to ArrayExpress (Data Citation 1). 15 samples were genotyped at both centres (Supplementary Table 4).

### Genotype calling

Genotypes were called from the CEL files using Affymetrix Power Tools (APT) version 1.14.2, and its BRLMM-P algorithm^24^. This algorithm uses information from all the CEL files as well as priors on the position and scatter of the clusters used to call genotypes. All the CEL files reported in this Descriptor were analysed together, as well as with a number of other inbred and outbred rats so as to improve the quality of the calls.

APT was run with the following command line:

apt-probeset-genotype
--cdf-file RATDIVm520813.CDF
--read-models-brlmmp models.txt
--cel-files celfilelist.txt
--a quant-norm.sketch=50000,pm-only,brlmm-p.CM=1.bins=100.mix=1.bic=2.HARD=3.SB=0.75.KX=0.2.KH=0.3.KXX=-0.1.KAH=-0.1.KHB=-0.1.transform=MVA.AAM=2.0.BBM=-2.0.AAV=0.06.BBV=0.06.ABV=0.06.copyqc=0.00000.wobble=0.05.CSepThr=4.CSepPen=0.1.KYAH=-0.05.KYHB=-0.05.KYAB=-0.1.AAY=10.5.ABY=11.BBY=10.5.copytype=-1.clustertype=2.ocean=0.00001.MS=0.1.hints=1.CP=16.Hok=1
--summaries
--select-probes
--write-models
--no-gender-force
--out-dir RESULTS

### Positioning the RATDIV SNPs on the Rnor5.0 assembly

The sequences targeted by the probes on the array were mapped to the latest reference genome (Rnor5.0) using BLAST^25^ and requiring an exact match of all 33 nucleotides to the plus or minus strand.

### Selection of a subset of high quality SNPs for haplotype reconstruction

**Figure 3:**
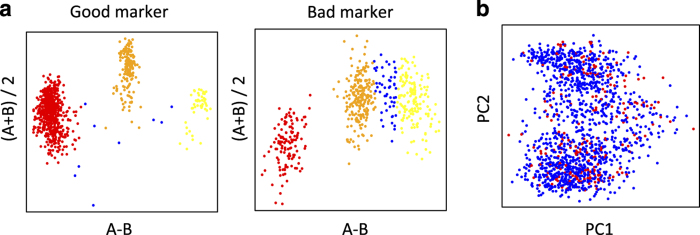
Genotyping quality control. (**a**) Genotyping samples shown in the hybridization signal space (A−B; (A+B/2)) used by BRLMM-P for calling genotypes. A and B are the raw hybridization signals in the CEL files. Each dot represents a sample. Homozygote calls are coloured in red and yellow, heterozygote calls in orange and uncalled samples in blue. Only markers showing three large, well-resolved clusters with few uncalled samples were selected for haplotype reconstruction. (**b**) Principal component analysis (PCA) of the genotype calls. Each dot is a sample. The position of the samples on the first two principal components. Those samples genotyped at CNG are coloured in blue, those genotyped at the MDC in red. The genotypes were encoded as 0,1,2 for this analysis, missing values were replaced by the minor allele frequency of the SNP, and the PCA was carried out without centering or scaling the genotypes.

A subset of 262,052 high-quality, informative markers were selected to reconstruct the genome of each HS rat as a mosaic of the founder genomes. This was motivated by the need for high-quality progenitor genotypes in order to ensure accurate haplotype reconstruction. We now list the filters used to select those markers. First, we kept only markers for which the RATDIV target sequence mapped perfectly and uniquely to Rnor5.0 (761,333 markers left). We excluded those SNPs that were within 13 bp of an indel (754,843 left). To apply the next filters, we used the reference sequence (BN) in place of the sequence and genotypes of the BN progenitor. This decision was made because the quality of the DNA available from that progenitor was poor. However, we only observed 2,892 genotype differences between the reference sequence and the sequence of the BN progenitor generated by us. We went on to exclude those SNPs for which the array-based or the sequence-based genotype of any of the progenitors was heterozygous or missing; we also excluded markers which were monomorphic across the 8 progenitors (490,147 markers left); we filtered out SNPs with a call rate lower than 0.99 (0.95 for markers on the X chromosome), those giving rise to 4 or more mendelian inconsistencies (calculated using the genotyped of 100 parents of HS rats, data not reported here), as well as those with fewer than 9 homozygous calls of each type (AA and BB) and 3 heterozygous calls. Also excluded were those markers with a ‘heterozygous offset’ lower than -0.05 or a Fisher linear discriminant value lower than 6 (as recommended by Affymetrix technical support, personal communication). Figure 3a illustrates a good quality SNP and one of poor quality. 262,052 markers passed all the filters. The average spacing between these SNPs is 12.5 kb. These SNPs are high-quality and informative, and were used to reconstruct the progenitor haplotypes in the HS rats using the software HAPPY.

Of these, 222,655 were used for the analysis reported in ref. 4, where SNP selection was based on alignment of the RATDIV probes and sequence data to Rnor3.4.

### Haplotype reconstruction in the outbred rats

The methods used to generate haplotype dosages (derived data) are detailed in ref. 4. Briefly, we used the R HAPPY package^7^ (www.well.ox.ac.uk/happy) to calculate descent probabilities from the 8 progenitors for each outbred rats at each of 262,052 inter-marker intervals and then averaged these probabilities over 90-kb windows, such that we eventually worked with 24,616 probability matrices.

### Whole genome shotgun sequencing of the eight progenitors

We generated DNA libraries from genomic DNA samples of the original rats that were used to create the Heterogeneous Stock (progenitors). The libraries were generated using standard protocols (Life Technologies) and had a median insert size of between 109 and 196 bp. All libraries were sequenced with fragment (50 bp) and paired-end (50+35 bp) runs using the next-generation sequencing (NGS) platform SOLiD 4 and SOLiD 5500 sequencers to a depth of at least 22x base coverage for each of the eight HS progenitors.

### Sequence alignment

The sequencing reads were mapped to the rat reference assembly Rnor3.4 using BWA^26^ v0.5.9 with parameters -c –l 25 –k 2 –n 10. They were aligned against the Rnor5.0 assembly using BWA 0.5.10 software with default parameters; the pairing of paired-reads was done using ‘sample’ option of BWA. Alignments from different libraries of the same HS progenitor were combined into a single BAM file (Supplementary Table 5).

### Variant calling

Variant calling was performed independently for each progenitor. SNPs and short insertions and deletions (indels) were called using different pipelines for the reads mapped to the Rnor3.4 assembly and those mapped to Rnor5.0. We start by presenting the former.

SNPs and short indels (<10 bp) were called using a modified Samtools^27^ pipeline: only unambiguously mapped reads were used to prevent false-positive that can be scored between paralogous sequences. Sites with coverage below 4 or over 2000 mostly represent NGS-inaccessible and repetitive sequences that are collapsed in genome reference, respectively, and were excluded from SNP/indel calling. Read bases with base-quality below 30 were considered unreliable and, thus, ignored during variant calling. Duplicate reads starting at the same position and mapped to the same strand as another read were discarded as likely PCR artefacts. Each of the called alleles had to be supported by at least one read where the variant mapped within the seed part of the read (first 25 bases). Non-reference alleles called with fewer than 3 reads were treated as missing genotypes. Variable sites with more than 2 alleles within one founder were set to missing. We considered the remaining variants to be homozygous non-reference alleles (frequency of non-reference call>2/3) or heterozygous alleles (frequency between 1/3 and 2/3).

Calling of SNPs and short indels based on alignments to Rnor5.0 was performed using GATK toolkit^28^. Only polymorphisms with scores 50 and above, with homozygous and different alleles (polymorphic among HS progenitor rats) were used. Prediction of the functional effect of each variant was performed by Variant Effect Predictor tool VEP 2.1 tool^29^.

We called copy number variants on both assemblies by using depth-of-coverage approach implemented in DWAC-Seq v. 0.56 (https://github.com/Vityay/DWAC-Seq) using default parameters. Structural variants (SVs) were called using discordant pair mapping implemented in 1-2-3-SV v. 1.0 (https://github.com/Vityay/1-2-3-SV), requiring unambiguous mapping of both paired tags and at least 4 tag pairs per SV event. SV calls from these tools were merged.

### False positive and false negative rates

The BAC and next-generation sequencing data for the LE/Stm discussed in the following paragraph are not included in this descriptor but are publicly available (see ref. 4).

The genome of the LE/Stm strain was sequenced on the SOLiD platform in the same way as the NIH-HS strains and analysed using the same bioinformatics pipeline (that for assembly Rnor3.4). Thirteen BACs from that same strain were sequenced using capillary methods, assembled and manually edited, producing a total of 2.1 Mb finished sequence. False positive and false negative rates were estimated from 1.9 Mb of genome sequence syntenic between BACs and genome assembly, excluding low quality BAC sequence (as defined by the BAC finishing team) and inaccessible regions (as defined above). False positive and false negative rates within this 1.9 Mb were estimated from the discordance between the BAC and NGS allele calls.

The false positive rate was independently investigated by PCR-based resequencing of a subset of variants chosen at random among those segregating between the HS progenitors. 96 SNPs and 96 indels were checked. Oligonucleotide primers were selected to amplify 300 bp fragments around the candidate polymorphism. When amplification was successful (SNPs: 84, indels: 80), amplicons were sequenced on an Applied Biosystems ABI 3730XL sequencer using Big-Dye terminator and analysed with Polyphred software manually.

184 copy number variants and structural variants were selected. PCR primers were designed so that the presence or absence (depending on the variation type) of a PCR product could confirm the presence of the variation. After PCR, samples were run on agarose gel and analysed manually. Of the 184 amplicons, 93 gave a PCR product in at least one of the progenitors.

The false positive and negative rates are presented in Table 2 and discussed in the technical validation section.

### Definition of NGS-accessible genome

In order to estimate the missing portion of variation that we failed to access with current NGS sequencing technology, we evaluated effective genome coverage of our NGS data. We defined inaccessible regions of the HS rat genomes as was done for mouse genomes^30^. A base was considered as accessible if it did not overlap simple, tandem repeats or low complexity sequence (defined by Dust, source: Ensembl release 66; http://www.ensembl.org), was not covered by more than 150 reads, and average mapping quality was at least 40. Nucleotide positions within 15 bp of indels were also considered as inaccessible for SNP calling.

## Data Records

The Rat Genome Sequencing and Mapping Consortium generated the data.

### Data record 1

Phenotypes of the HS rats, and experimental covariates. measures.txt is a tab-delimited text file with values for 195 phenotypes and 21 covariates that may affect the phenotypic measurements, for a total of 2,006 outbred rats. All phenotypes and covariates are described in Supplementary Table 1. Missing values are encoded as NA. measures.txt was submitted as supplementary file to ArrayExpress (Data Citation 1) as well as figshare (Data Citation 2).

### Data record 2

Genotypes of the HS rats and the progenitors of the Stock. The genotypes file is a tab-delimited text file with genotypes for 1,407 outbred rats, 15 of which were genotyped twice (once in each genotyping centre, MDC and CNG, see Methods), as well as the eight progenitors. It includes genotypes for all 805,399 markers on the RATDIV array. Genotypes are encoded as 0 (reference homozygote), 1 (heterozygote), 2 (non-reference homozygote). Missing values are encoded as NA. The genotypes file E-MTAB-2332.processed.1.zip is available from the processed file download link in ArrayExpress experiment accession E-MTAB-2332 (Data Citation 1).

### Data record 3

List of high-quality markers. markers_selected_for_Rn50.txt is a tab-delimited text file listing the 262,052 markers selected for haplotype reconstruction based. Each marker is identified by its RATDIV ID (‘Rn34_’), which refers to its initial position on Rnor3.4. The position of the marker on Rnor5.0 is indicated. This file was submitted as a supplementary file to ArrayExpress (Data Citation 1).

### Data record 4

Haplotype dosages for the HS rats. haplotype_dosages_Rnor50.tar.gz was obtained by reconstructing the genome of each HS rat as a mosaic of the progenitor genomes using the piece of software HAPPY^7^. The reconstruction is based on the markers listed in markers_selected_for_Rn50.txt. The file corresponds to HAPPY’s so called genome cache, a collection of R objects (‘.RData’) that are best utilised with the R HAPPY package (see www.well.ox.ac.uk/happy). The genome cache contains the probabilities to be descended from each of the eight progenitors (haplotype dosages), for each HS rat and at each locus in the genome. Haplotype dosages are available for an additive and a full model^7^. At a given locus, the probabilities are presented in an N x s matrix, where N is the number of HS rats (1,407+15 rats that were genotyped twice) and s is the number of progenitors (8) for the additive model, and the number of unique pairs of progenitors (32) for the full model. The IDs of the columns are stored in the strains.RData object, the IDs of the rows in the subjects.RData object. Each matrix is an R object called by the name of the marker at the leftmost position of the interval^4,7^ (there are only 24,616 matrices in the genome cache because the haplotype dosages were averaged over 90 kb windows as explained in ref. 4). The HS rats are identified by the name of the corresponding CEL file in the genome cache (i.e., in subjects.RData). The correspondence with the IDs used throughout the other data reported in this Descriptor is available from Supplementary Table 4 and from the metadata submitted to ArrayExpress (Data Citation 1), as is the ‘duplicate’ status of each rat. haplotype_dosages_Rnor50.tar.gz was submitted to figshare (Data Citation 2).

### Data record 5

Sequence variants for the progenitors. HS_SNPs_Rn34.txt.gz, HS_Indels_Rn34.txt.gz, HS_SVs_Rn34.txt.gz, HS_SNPs_Indels_Rn50.txt.gz and HSLE_SVs_Rn50.txt.gz are tab-delimited text files containing the SNPs, indels, and structural variants (SVs) called for each of the eight progenitors (plus the rat strain Long Evans (LE) for HSLE_SVs_Rn50.txt.gz). The assembly against which the sequencing reads were aligned for variant calling is indicated in the name of the file. Missing values are encoded as N in the SNPs and indels related files, while only positive calls are made for the structural variants (SVs). In HS_SVs_Rn34.txt.gz and HSLE_SVs_Rn50.txt.gz, a deletion is annotated as Deletion(RD), a duplication as Duplication(RD), an inversion as Inverted_pair(RP), a tandem duplication as Everted_pair(RP) and a deletion Distant_tags(RP), where RD refers to a method for calling variants based on read depth, and RP a method based on orientation and spacing of read pairs (see methods). For those variants called by the RP method, the number of read pairs supporting the call in indicated in brackets for each progenitor. For variants called with the RD method, the ratio between the number of reads in the progenitor and the number of reads in a reference (BN/Mcwi ‘Eve’ rat sequence) is indicated for each progenitor. Those variants supported by at least 4 pairs (RP) or with copy number change greater than 25% were included in the file.

All files were submitted as supplementary files to ArrayExpress (Data Citation 1).

### Data record 6

Accessible genome. *_genome_accessibility_Rn34.bed.gz and *_genome_accessibility_Rn50.bed.gz where * takes values in {ACI, BN, BUF, F344, M520, MR, WKY, WN} are bed files with the intervals deemed accessible for next –generation sequencing. They were calculated for both assemblies as indicated in the name of the file. All files were submitted as supplementary files to ArrayExpress (Data Citation 1).

### Data record 7

Progenitor SNPs and indels reformatted in an R object suitable for merge analysis. merge_factors.RData is an R object which when loaded into an R session is a list with two components: 1) a data.frame with position, genotypes for all SNPs and indels called on Rnor5.0 for the eight progenitors, and corresponding strain distribution pattern (i.e., an indication of which progenitors share the same allele) and 2) a list with as many unique elements as unique strain distribution patterns, and each element a matrix by which the appropriate matrix of haplotype dosages should be multiplied for merge analysis (see refs 4,^8^ for more information on the merge analysis). merge_factors.RData was submitted to figshare (Data Citation 2).

### Data record 8

Haplotype dosages and imputed genotypes at all sequence variants for the HS rats. The file HS.hdf5 is an HDF5 file (http://www.hdfgroup.org/HDF5/) containing two elements: 1) additive haplotype dosages stored in an 1,422×8×24,616 array and 2) imputed genotypes for 1,422 observation (1,407 rats+15 duplicates) at 543,4692 SNPs and indels. Information about the rats, the variants, etc. is also stored in the HDF5 file, which was submitted as supplementary files to ArrayExpress (Data Citation 1).

## Technical Validation

### Comparison of variants calls from NGS and Sanger sequencing to estimate the false positive and false negative rates, and confirmation by PCR-based re-sequencing

False positive and negative rates were estimated by sequencing 2.1 Mb of LE/Stm BAC libraries by both the next generation sequencing (NGS) pipeline used for the HS progenitors and Sanger sequencing (gold standard). Table 2 shows rather high false negative rates but small false positive rates.

The low false positive rates were confirmed by PCR-resequencing. All of the 84 SNPs and 80 indels for which amplification was successful were confirmed. Of the 93 structural variants (SVs) that gave a PCR product, a group of 39 variants that were predicted SVs in the NIH-HS founders, were also confirmed by PCR in the reference substrain BN/NHsdMcwi, indicating that these are probably assembly errors in the reference genome Rnor3.4. Of the remaining 54 variants, 53 gave a banding pattern in agreement with the NGS calls.

### Concordance between technical duplicates and principal component analysis (PCA) to investigate whether samples genotyped at one centre are different from samples genotyped at the other centre

15 HS rats were genotyped at both centres (CNG and MDC) following DNA extraction at MDC. Using the subset of 262,052 high-quality SNPs, we investigated the concordance between these technical replicates. The average concordance across the 15 duplicates is 0.997, showing that the genotypes were used for subsequent analyses are very reliable.

A PCA was carried out using the genotypes at all SNPs on the RATDIV array. Figure 3b shows that there is no batch effect attributable to the genotyping centre.

## Usage Notes

Our recent publication^4^ illustrates how our data can be used and we refer the reader to it for more information on how we analysed our data and interpreted the results.

Figure 2b briefly recapitulates which data are combined for genetic mapping, and merge analysis.

A brief usage note on how to perform merge analysis is provided in Supplementary Information 1.

## Additional information

**How to cite this article:** Baud, A. *et al.* Genomes and phenomes of a population of outbred rats and its progenitors. *Sci. Data* 1:140011 doi: 10.1038/sdata.2014.11 (2014).

## Supplementary Material



Supplementary Information

Supplementary Table 1

Supplementary Table 2

Supplementary Table 3

Supplementary Table 4

Supplementary Table 5

## Figures and Tables

**Table 1:** Phenotyping pipeline.

**Phenotype**	**Disease model**	**Number of measures**	**Age (weeks)**
Coat colour		1	7
Wound healing		1	7 and 17
Fear related behaviours	Anxiety	48	8 to 10
Glucose tolerance	Type II diabetes	6	11
Cardiovascular function	Hypertension	2	12
Basal hematology		32	13
Basal immunology		34	13
**Immunization with myelin oligodendrocyte protein**			13
Serum biochemistry		15	17
Arterial elastic lamina ruptures		6	17
Renal agenesis		1	17
Body weight	Obesity	6	8, 9, 11, 13, 14, and 17
Bone mass and strength	Osteoporosis	43	17

**Table 2:** Comparison of variants calls from NGS and Sanger sequencing.

**Call type**	**True positives**	**False positives (FPR)**	**False negatives (FNR)**
SNPs	2290	63 (2.7%)	475 (17.2%)
Indels	178	4 (2.2%)	126 (41.4%)
Structural variants	10	2 (16.7%)	19 (65.5%)

## References

[d1] ArrayExpress The Rat Genome Sequencing and Mapping Consortium 2014 E-MTAB-2332

[d2] Figshare The Rat Genome Sequencing and Mapping Consortium 2014 http://dx.doi.org/10.6084/m9.figshare.943485

