# Supplementary material for: Genomes and phenomes of a population of outbred rats and its progenitors
Source: Sci Data. 2014 Jun 10;1:140011. doi: 10.1038/sdata.2014.11 (PMC4381735; doi:10.1038/sdata.2014.11)
Supplement: Supplementary Information [file sdata201411-s2.pdf]

```
# usage note for "Genomes and phenomes of a population of outbred rats and its progenitors", Baud et al., Nature Scientific Data 2014
# note by Amelie Baud (abaud@ebi.ac.uk)
# also see "Combined sequence-based and genetic mapping analysis of complex traits in outbred rats", Rat Genome Sequencing and Mapping Consortium, Nature Genetics 45, 767-75 (2013) for more information and references

# one needs to account for different degrees of relatedness in the HS when testing for association between phenotype and haplotype/genotype, which can be done using mixed models (or other methods)
# this note focuses on the test of whether a sequence variant explains phenotypic variation at a QTL significantly better than the haplotypes do ("merge analysis"). It only provides the few lines of R code central to merge analysis.
# this code needs to be integrated with the method chosen by the user to account for relatedness

# this code uses as input the phenotype data, the user's HAPPY genome cache, and the R object merge.data that can be loaded into R from merge_factors.RData (available from figshare)
# mhaplotype is a N x 8 HAPPY probability matrix (i.e. haplotype dosages) where N is the number of outbred rats included in the model and 8 the number of progenitors
# sdp is the strain distribution pattern of the variant that is going to be tested. The strain distribution pattern of all the SNPs and indels is available from the R object merge.data
# Note: the variant has to be within the interval corresponding to the HAPPY probability matrix! The boundaries of the interval are given by: on the left, the position of the marker whose name ("Rn34_…") is that of the HAPPY probability matrix in the genome cache; on the right, the position of the next marker in the genome cache. Whenever an interval is much wider than 90kb, the right boundary is imprecise. However, these are intervals where the haplotype reconstruction may have been imprecise in the first place, so the results should be examined carefully.


msdp = merge.data[[2]][[sdp]]
mmerged = mhaplotype %*% msdp

#null model
#X_mat is the matrix of covariates (it may need to include a vector of 1 for the intercept in the mixed model framework)
fit_haplotype = lm (y ~ X_mat + mhaplotype)

#alternative model
fit_merged = lm (y~ X_mat + mmerged)

#test
a = anova( fit_haplotype, fit_merged)
```

```
# Note: merge_matrix is essentially a matrix of genotypes.
Therefore, one can also use the haplotype and genotypes dosages
(imputed variants) available from ArrayExpress in the file HS.hdf5
to carry out this test.
```
